# Recurrent but Short-Lived Duplications of Centromeric Proteins in Holocentric *Caenorhabditis* Species

**DOI:** 10.1093/molbev/msac206

**Published:** 2022-09-29

**Authors:** Lews Caro, Pravrutha Raman, Florian A Steiner, Michael Ailion, Harmit S Malik

**Affiliations:** Molecular and Cellular Biology Program, University of Washington, Seattle, WA 98195, USA; Department of Biochemistry, University of Washington, Seattle, WA 98195, USA; Division of Basic Sciences, Fred Hutchinson Cancer Center, Seattle, WA 98109, USA; Department of Molecular Biology and Cellular Biology, Section of Biology, Faculty of Sciences, University of Geneva, Geneva, Switzerland; Molecular and Cellular Biology Program, University of Washington, Seattle, WA 98195, USA; Department of Biochemistry, University of Washington, Seattle, WA 98195, USA; Division of Basic Sciences, Fred Hutchinson Cancer Center, Seattle, WA 98109, USA; Howard Hughes Medical Institute, Fred Hutchinson Cancer Center, Seattle, WA 98109, USA

**Keywords:** centromeric histone, gene duplication, protein motifs, kinetochore

## Abstract

Centromeric histones (CenH3s) are essential for chromosome inheritance during cell division in most eukaryotes. CenH3 genes have rapidly evolved and undergone repeated gene duplications and diversification in many plant and animal species. In *Caenorhabditis* species, two independent duplications of *CenH3* (named *hcp-3* for *HoloCentric chromosome-binding Protein 3*) were previously identified in *C. elegans* and *C. remanei*. Using phylogenomic analyses in 32 *Caenorhabditis* species, we find strict retention of the ancestral *hcp-3* gene and 10 independent duplications. Most *hcp-3L* (*hcp-3-like*) paralogs are only found in 1–2 species, are expressed in both males and females/hermaphrodites, and encode histone fold domains with 69–100% identity to ancestral *hcp-3*. We identified novel *N*-terminal protein motifs, including putative kinetochore protein-interacting motifs and a potential separase cleavage site, which are well conserved across *Caenorhabditis* HCP-3 proteins. Other *N*-terminal motifs vary in their retention across paralogs or species, revealing potential subfunctionalization or functional loss following duplication. An *N*-terminal extension in the *hcp-3L* gene of *C. afra* revealed an unprecedented protein fusion, where *hcp-3L* fused to duplicated segments from *hcp-4* (nematode CENP-C). By extending our analyses beyond *CenH3*, we found gene duplications of six inner and outer kinetochore genes in *Caenorhabditis*, which appear to have been retained independent of *hcp-3* duplications. Our findings suggest that centromeric protein duplications occur frequently in *Caenorhabditis* nematodes, are selectively retained for short evolutionary periods, then degenerate or are lost entirely. We hypothesize that unique challenges associated with holocentricity in *Caenorhabditis* may lead to this rapid “revolving door” of kinetochore protein paralogs.

## Introduction

The faithful inheritance of genetic material is indispensable for all life. In most eukaryotes, faithful inheritance of chromosomes relies on the centromeric histone H3 variant (*CenH3*) to attach chromosomes to microtubules. CenH3 acts both as a structural component of the multi-subunit complex that links chromosomes to microtubules for segregation and as the epigenetic mark that defines and maintains the centromeric location(s) on chromosomes (Allshire and Karpen 2008; De Wulf and Earnshaw 2008; Fukagawa and Earnshaw 2014; McKinley and Cheeseman 2016; Ali-Ahmad and Sekulić 2020; Mellone and Fachinetti 2021). CenH3 is critical for chromosome segregation during mitosis and meiosis. Mutations or misregulation of CenH3 have severe consequences for fertility and viability in many species (Stoler et al. 1995; Buchwitz et al. 1999; Howman et al. 2000; Blower and Karpen 2001). CenH3 would therefore be expected to be conserved across eukaryotes and expected to evolve under strong evolutionary constraints to maintain functionality.

Despite this expectation for strong conservation, *CenH3* genes have rapidly evolved in animal and plant species (Malik and Henikoff 2001; Talbert et al. 2004; Schueler et al. 2010). This rapid evolution is hypothesized to result from a unique genetic conflict that stems from asymmetric female meiosis in animals and plants, in which only one of four meiotic products gets selected to be included in the oocyte nucleus. As a result of this bottleneck, chromosomes compete for inclusion into the egg in a process termed “centromere drive” (Henikoff et al. 2001; Malik 2009; Schueler et al. 2010; Lampson and Black 2017). This competition favors changes in centromeric DNA that result in over-recruitment of centromeric proteins (Chmátal et al. 2014; Akera et al. 2017; Iwata-Otsubo et al. 2017). Conversely, genes encoding centromeric proteins evolve rapidly to suppress the “selfish advantage” of cheating centromeres to restore parity and ameliorate the deleterious effects of centromere-drive (Finseth et al. 2021; Kumon et al. 2021). Thus, in many animal and plant species, CenH3 proteins evolve rapidly despite being essential for faithful chromosome segregation.

CenH3 proteins can also function differently during meiotic and mitotic segregations. Some plant *CenH3* mutants only show defects during meiosis, but not mitosis (Lermontova et al. 2011; Ravi et al. 2011; Schubert et al. 2014). Conflicting evolutionary selective pressures on *CenH3* between these functions (e.g., mitotic vs. meiotic, conserved vs. rapidly evolving) could be resolved by gene duplication, which allows the duplicate (paralog) and ancestral genes to specialize for different functions (Hittinger and Carroll 2007; Des Marais and Rausher 2008; Gallach and Betrán 2011). Indeed, *CenH3* genes have also undergone repeated gene duplications not just in plants but also in several animal species including cows, fruit flies, mosquitoes, and nematodes (Li and Huang 2008; Zedek and Bureš 2016; Kursel and Malik 2017; Ishii et al. 2020; Kursel et al. 2020, 2021; Despot-Slade et al. 2021; Elisafenko et al. 2021). Cytological evidence in *Drosophila virilis* suggests that divergent *CenH3* paralogs can acquire separate, tissue-specific functions (Kursel et al. 2021).

Although *CenH3* has undergone duplication and diversification in *Drosophila* and mosquito species, four orders of insects have completely lost *CenH3* (Drinnenberg et al. 2014). *CenH3* loss appears to correlate with transitions from monocentricity, in which centromeric determinants are concentrated in one genomic region, to holocentricity, in which centromeres are dispersed along the length of their chromosomes. Thus, holocentricity may impose unique selective pressures that shape the path of *CenH3* and kinetochore evolution (Marques and Pedrosa-Harand 2016; Cortes-Silva et al. 2020; Senaratne et al. 2022; Wang et al. 2022).

In contrast to holocentric insects that have lost *CenH3*, *CenH3* homologs are present in other holocentric animal and plant species (Drinnenberg et al. 2014). Moreover, several nematode clades encode duplications and diversification of *CenH3* genes (Despot-Slade et al. 2021). Holocentric chromosome segregation in nematodes has been best studied in *C. elegans*, which encodes two *CenH3* paralogs. The first of these to be characterized was *hcp-3*, which encodes a protein required for recruiting all other kinetochore proteins and is essential for embryonic mitotic divisions in *C. elegans* (Buchwitz et al. 1999; Oegema et al. 2001). However, HCP-3 appears to be dispensable for oocyte meiotic segregation (Monen et al. 2005). A second *CenH3* paralog in *C. elegans*, CPAR-1, shares high sequence similarity to HCP-3 in the histone fold domain (HFD) but is diverged in the *N*-terminal domain (Monen et al. 2015). Although CPAR-1 is enriched in meiotic chromosomes, it does not appear to localize to centromeres at all, and its precise function is not well understood (Gassmann et al. 2012; Monen et al. 2015). An independent *hcp-3* duplication occurred in a related species, *C. remanei* (Monen et al. 2015), but its function is also unknown. These previous studies left unclear whether *CenH3* duplications in *C. elegans* and *C. remanei* were unusually rare or typical of *Caenorhabditis* nematodes.

Faithful chromosome segregation in *C. elegans* relies not only on CenH3 alone but also on CenH3 interaction with HCP-4 (CENP-C in mammals) and KNL-2 to form the inner kinetochore. A predicted structured region of the HCP-3 *N*-terminal tail interacts with KNL-2 (de Groot et al. 2021; Prosée et al. 2021). This interaction is necessary for the establishment of centromeres in the hermaphrodite germline, prior to the first embryonic mitosis (Prosée et al. 2021). Identifying which HCP-3 residues are important for protein interactions has been challenging, owing to low sequence identity of CenH3 among species (de Groot et al. 2021; Prosée et al. 2021). Despite high sequence divergence of CenH3 *N*-terminal tails, *CenH3* evolution is likely constrained to maintain important protein–protein interaction interfaces (Malik et al. 2002; Maheshwari et al. 2015). Identifying these constraints may reveal insights into the molecular architecture of such interactions. Thus, a phylogenetic study of CenH3 and kinetochore protein evolution and duplication in *Caenorhabditis* nematodes would not only yield insights into the cadence of gene duplication and retention but also reveal functional constraints that would inform the molecular interactions that underlie the important function of chromosome segregation.

The growing collection of *Caenorhabditis* species and their genome sequences (Stevens et al. 2019) (unpublished genomes at http://caenorhabditis.org/) provides a rich dataset for identifying both the evolutionary trajectory and constraints of their *CenH3* genes. Taking advantage of this resource, we performed detailed phylogenomic analyses to understand the evolution of *CenH3* genes in *Caenorhabditis*. Our studies reveal that 13 out of 32 analyzed *Caenorhabditis* species encode two or more *CenH3* paralogs, which were the result of at least 10 independent duplication events. We confirm that these paralogs are expressed in both sexes in representative species. We identify novel, conserved protein motifs within the *N*-terminal domains of *Caenorhabditis* CenH3 proteins that are likely important for interactions with other kinetochore proteins and for centromere biology. Although some motifs are strictly retained, others display variable instances of loss and retention between ancestral and duplicate genes, revealing clues to their subfunctionalization. In a possible case of neofunctionalization, we find an unusual *CenH3* paralog in *C. afra* that encodes a CENP-C-CenH3 fusion protein. Extending our analyses beyond *CenH3*, we find independent duplications of other inner and outer kinetochore proteins, revealing a remarkable pace of diversification of the kinetochore within *Caenorhabditis* nematodes. Our analyses thus reveal an unusual “revolving door” of CenH3 protein duplications, with retention only over short evolutionary periods. This pattern contrasts with the strict, long-lived retention of *CenH3* paralogs seen in *Drosophila*, mosquito, plant, and even other holocentric nematode species (Maheshwari et al. 2015; Kursel and Malik 2017; Kursel et al. 2020, 2021, Despot-Slade et al. 2021). We hypothesize that this pattern may result from the unusual mechanisms of centromere establishment and inheritance in holocentric *Caenorhabditis* species.

## Results

### *hcp-3* Has Duplicated At Least Ten Independent Times in *Caenorhabditis*

**Fig. 1.**
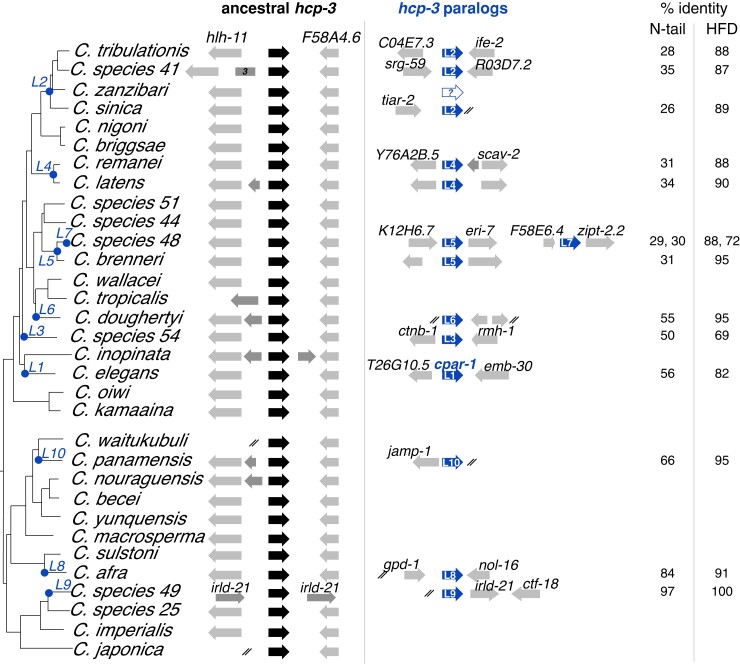
Ten independent *hcp-3* duplications in *Caenorhabditis* species. A schematic representation of ancestral centromeric histone genes (*hcp-3*, black) and their duplicates (*hcp-3L*, blue) are shown alongside a *Caenorhabditis* species tree (adapted from http://caenorhabditis.org). *hcp-3* duplication events are represented on the species tree with a blue dot and numbered *L1* through *L10*, with paralogs arising from independent duplications assigned different numbers. Genes in the syntenic neighborhood near *hcp-3* and *hcp-3L* are represented in gray and labeled with their orthologous gene names in *C. elegans*. In some cases, 1–3 genes were inserted between *hlh-11* and *F58A4.6* within the syntenic neighborhood of *hcp-3*. The white arrow with a question mark represents a possible loss of *hcp-3L2* in *C. zanzibari*. Ends of genomic scaffolds are denoted with two slashes. On the right, we show percent amino acid identities between the paralog and ancestral *hcp-3* of each species (in the *N*-terminal tail or HFD).

Global efforts to isolate and sequence *Caenorhabditis* species have recently resulted in several well-assembled genomes from highly diverged species (Stevens et al. 2019) (unpublished genomes at http://caenorhabditis.org/). We used this resource for phylogenomic analyses of *CenH3* evolution. We used *C. elegans* HCP-3 as a query for tBLASTn searches against genome sequences from 32 *Caenorhabditis* species (Altschul et al. 1990, 1997; Stevens et al. 2019; http://caenorhabditis.org/) to identify all *hcp-3* homolog (*hcp-3*-like) genes (supplementary data S1, Supplementary Material online) and their syntenic location (surrounding genes) (fig. 1). Core histone H3 and H3 variant genes were also obtained in these analyses but were easily distinguished from *hcp-3* homologs because of their high similarity to each other. Since our focus was on putative *hcp-3* orthologs and paralogs, we ignored both highly conserved core histone H3 and H3 variant proteins, as well as species-specific instances of highly diverged H3-like genes such as *F20D6.9* (also referred to as *D6H3*) from *C. elegans* (Henikoff et al. 2000; Delaney et al. 2018).

Unlike in holocentric insects (Drinnenberg et al. 2014), we found that *hcp-3* orthologs are strictly retained in all *Caenorhabditis* species. In 28 of 32 species, they are found in shared syntenic locations, between genes homologous to *C. elegans hlh-11* and *F58A4.6* (fig. 1). In three of the four remaining species, at least partial synteny is maintained downstream of *hcp-3* (genes *F58A4.6*, *pri-1*, and *bbs-4*) whereas upstream synteny is either not maintained (in *C. tropicalis*) or cannot be discerned due to short genomic scaffolds (*C. waitukubuli* and *C. japonica*, fig. 1). Only *C. species 49* (*C. sp49*) lacks an *hcp-3* gene in this shared syntenic locus. Based on its presence in the ancestral locus in its sister species *C. sp25* and all other species, we infer that this movement of *hcp-3* is specific to *C. sp49*. *C. sp49* encodes two *CenH3* paralogs, both found in new syntenic loci that are not shared with sister species. We arbitrarily assign one homolog as *hcp-3* and the other as *hcp-3L9* (further explained below).

**Fig. 2.**
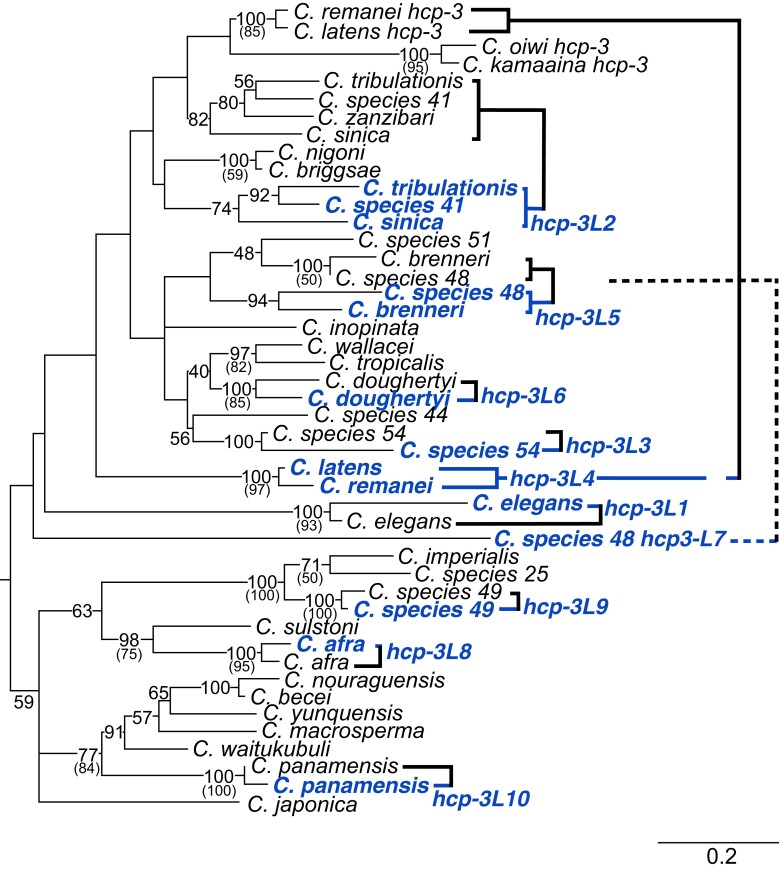
Phylogenetic analysis of *hcp-3* and *hcp-3L* genes from *Caenorhabditis* species. A maximum likelihood tree of a DNA, codon-based alignment of the HFD of ancestral *hcp-3* (black) and *hcp-3* paralogs (blue) is shown. Bootstrap values of 40 and above are indicated. Bootstrap values in parentheses are from corresponding nodes from a maximum likelihood tree based on an amino acid alignment of the HFD (see supplementary fig. S2, Supplementary Material online). In all except a few instances, the nucleotide and amino acid tree are in agreement, with higher bootstrap support observed in the nucleotide tree. For the exceptions (nodes representing *hcp-3* or *hcp-3L2* in *C. tribulationis*, *C. sinica*, *C. sp41*, and *C. zanzibari*, and the node representing *hcp-3* in *C. nouraguensis*, *C. becei*, and *C. macrosperma*), bootstraps values were not included here since they were lower in the amino acid tree and because they do not alter conclusions from the nucleotide tree. A scale bar (branch lengths, substitutions per site) is shown at the bottom-right. On the right, thick lines show *hcp-3* paralogs from same species, the dashed line shows the second duplicate found in *C. species 48*.

In addition to *hcp-3* orthologs, we found that 13 out of 32 examined species encode at least one additional *hcp-3*-like sequence. We refer to these paralogs as “*hcp-3L*” genes (for *hcp-3Like*) (fig. 1). These *hcp-3L* genes include previously reported *hcp-3* duplications in *C. remanei* and *C. elegans* (Monen et al. 2005, 2015), which we refer to as *hcp-3L4* and *cpar-1* (as previously named, also referred to as *hcp-3L1* in figs. 1 and 2), respectively. We also identified one additional *hcp-3L* paralog in *C. tribulationis*, *C. sp41*, *C. sinica*, *C. latens*, *C. brenneri*, *C. doughertyi*, *C. sp54*, *C. panamensis*, *C. afra*, and *C. sp49*, and two, independent *hcp-3L* paralogs in *C. sp48*. In most cases, *hcp-3L* paralogs shared identical exon–intron structure as their orthologs. However, we also observed a few instances of intron losses and gains in *hcp-3* or *hcp-3L* genes (supplementary fig. S1, Supplementary Material online). Such partial intron losses have been observed previously in plants (Roy and Penny 2007), fungi (Nielsen et al. 2004), and in *Caenorhabditis* species (Robertson 1998; Cho et al. 2004; Kiontke et al. 2004) and are thought to result from partial retrotransposition, in which cDNA partially replaced the genomic locus.

All *hcp-3* and *hcp-3L* genes encode proteins with conserved HFDs (see supplementary data, Supplementary Material online), which are between 69% and 100% identical to the HFD of HCP-3 from the same species (fig. 1). In contrast, their *N*-terminal domains show high divergence from HCP-3 orthologs (26–97% identical, fig. 1). This pattern is consistent with overall trends of *CenH3* evolution, where the HFDs are more evolutionarily constrained due to interactions with other histones, whereas the *N*-terminal domains can be so divergent that they cannot even be reliably aligned across different lineages (Malik and Henikoff 2001).

We next used a combination of syntenic and phylogenetic analyses to determine whether *hcp-3L* paralogs were shared between different species, which would indicate their functional co-retention with *hcp-3* orthologs for long evolutionary periods. The highly divergent *N*-terminal tail sequences of *hcp-3* and their paralogs cannot be reliably aligned and could distort our interpretations, so our phylogenies are based on HFD alignments. We first used the amino acid sequences for a maximum-likelihood phylogenetic analysis (supplementary fig. S2, Supplementary Material online). We found that the protein-based phylogeny suffered from poor resolution, was unable to resolve most of the important branches and groupings of interest, and was even incongruous with the well-accepted *Caenorhadbitis* phylogeny. Therefore, we built a maximum-likelihood phylogenetic tree using a codon-based alignment of the conserved HFD cDNA sequence (fig. 2). This phylogeny is much better resolved especially at shallow nodes (both phylogenies suffer from lack of resolution at deeply branching nodes) and largely agrees with our findings from the shared synteny analyses. For example, both syntenic and phylogenetic analyses suggest that the duplication that gave rise to *hcp-3L4* occurred prior to the common ancestor of *C. latens* and *C. remanei* (fig. 2). Similarly, we can infer that *hcp-3L5* duplicated in the common ancestor of *C. sp48* and *C. brenneri*. In contrast, the *hcp-3L* paralogs in *C. doughertyi*, *C. sp54*, *C. elegans*, *C. panamensis*, *C. afra*, *C. sp49*, and the additional *hcp-3L* paralog in *C. sp48* each arose via seven independent duplications (fig. 2). In each of these seven species, the *hcp-3L* paralogs are present in unique genomic locations (fig. 1) and typically group most closely with *hcp-3* orthologs from the same species (fig. 2).

The only discrepancy between the synteny and phylogenetic analyses was for *hcp-3L2* genes found in *C. tribulationis*, *C. sp41*, and *C. sinica*. These species are part of a group, with *C. sinica* believed to be an outgroup to *C. tribulationis*, *C. sp41*, and *C. zanzibari*. Different genomic locations of *hcp-3L* duplicates among *C. tribulationis*, *C. sp41*, and *C. sinica* (fig. 1) would suggest that the duplications are the result of independent duplication events although the small size of *C. sinica* genomic scaffolds leave its shared synteny status ambiguous. In contrast, our phylogenetic analyses group *hcp-3L* genes from these species together with a high degree of confidence (fig. 2), suggesting that *hcp-3L2* is the result of a single duplication event, followed by transposition of this gene to a new locus in *C. sinica*. We infer that the absence of *hcp-3L2* in *C. zanzibari* could be the result of gene loss although there is no evidence of *hcp-3L* loss in any other species. Another possibility is that *C. zanzibari* may be ancestral to *C. tribulationis* and *C. sinica* for the *hcp-3L* syntenic location, in contrast to the accepted species phylogeny, and may have never acquired a *hcp-3L* paralog. Recent studies have revealed widespread roles in diverse taxa for introgression and/or incomplete lineage sorting, leading to different genomic locations having vastly different evolutionary histories (Hobolth et al. 2011; Mailund et al. 2014; Ginsberg et al. 2019; Suvorov et al. 2022). Thus, it is formally possible that *C. zanzibari* never acquired *hcp-3L2*. However, based on the well-resolved species phylogeny of this quartet of species, we favor the first possibility that *C. zanzibari* acquired, then lost *hcp-3L2*. Therefore, our analyses reveal that *hcp-3* has duplicated at least ten independent times within *Caenorhabditis* species.

**Fig. 3.**
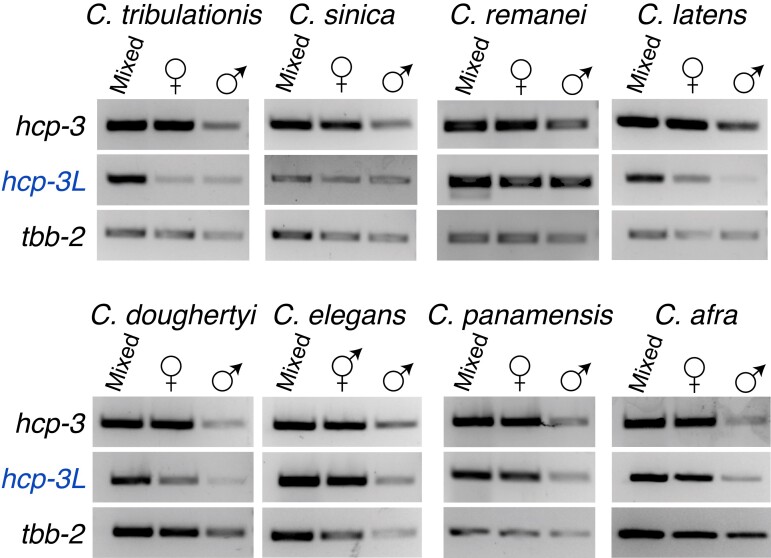
*hcp-3L* genes are expressed in both sexes in *Caenorhabditis* species. RT-PCR of ancestral *hcp-3* (top), *hcp-3L* (middle), or *tbb-2* (bottom; loading control) in species with *hcp-3* duplicates. RNA from a mixed worm population of various larval stages, L4 or young adult females/hermaphrodites or L4 or young adult males were used.

We examined the expression of *hcp-3* and *hcp-3L* genes across representative *Caenorhabditis* species. We used RT-PCR analyses using specific primers on template RNA collected from a mixed population of males and females or hermaphrodites at various larval stages (see Methods). All analyzed species expressed both ancestral and duplicate *hcp-3* genes (fig. 3; supplementary fig. S3, Supplementary Material online). We investigated whether *Caenorhabditis hcp-3L* genes have sex-restricted expression as is seen in some *Drosophila CenH3* paralogs (Kursel and Malik 2017). We performed RT-PCR on RNA collected from L4/young adult males or from L4/young adult hermaphrodites or females (these developmental stages capture both female and male meiosis). Unlike *Drosophila CenH3* paralogs, we did not find sex-restricted expression of any *hcp-3L* genes (fig. 3; supplementary fig. S3, Supplementary Material online); instead, they appear to be expressed in both sexes.

### Motif Retention and Loss in the *N*-terminal Region of HCP-3 and HCP-3L Proteins

Although CenH3 proteins all have a relatively conserved HFD, their *N*-terminal tails are often so divergent that they cannot be aligned nor even be considered homologous across different lineages (Malik and Henikoff 2001). Nevertheless, conserved motifs have been identified in the *N*-terminal tails of CenH3 proteins from many other lineages including *Drosophila*, mosquitos, and plants using alignment-independent approaches (Maheshwari et al. 2015; Kursel and Malik 2017; Kursel et al. 2020). These *N*-terminal tail motifs are often highly conserved within a lineage, but not conserved across different lineages. Although no such studies have been previously performed for the *Caenorhabditis* HCP-3 proteins, recent studies show that the *N*-terminal tail of *C. elegans* HCP-3 interacts with the inner kinetochore protein KNL-2 via a predicted structured region (de Groot et al. 2021; Prosée et al. 2021). This interaction between KNL-2 and HCP-3 is necessary for the establishment of centromeres in the hermaphrodite germline, prior to the first embryonic mitosis (Prosée et al. 2021).

**Fig. 4.**
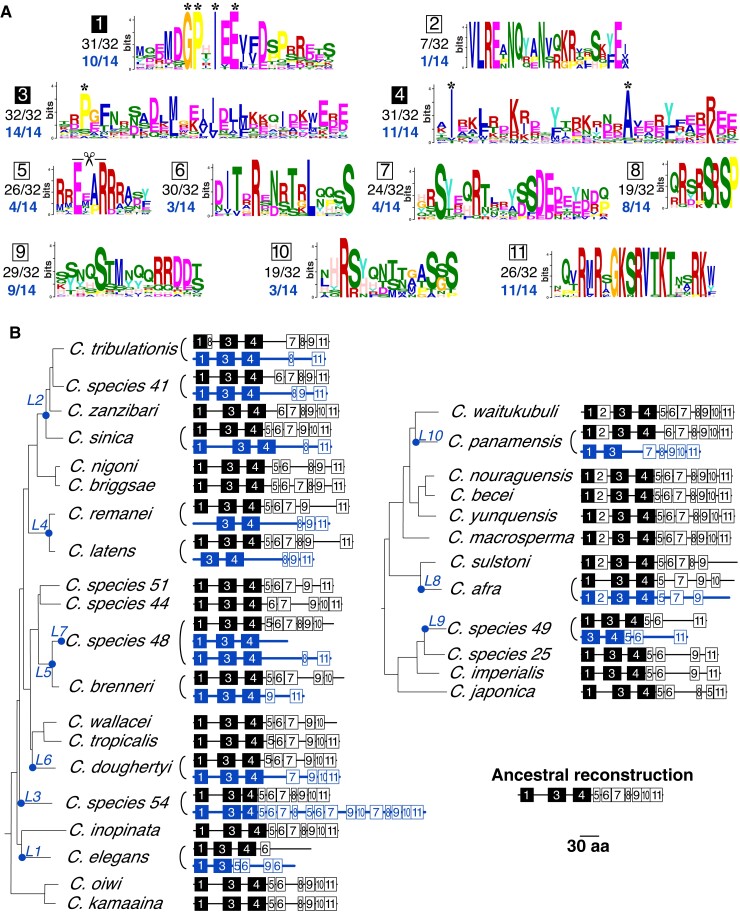
Differential retention of *N*-terminal tail motifs across HCP-3 and HCP-3L proteins encoded by *Caenorhabditis* species. (*A*) Logo plots of 11 protein motifs within HCP-3 *N*-terminal tails discovered from an analysis of *Caenorhabditis* species without duplications. Motifs 12 and 13 are C-terminal motifs (not shown, see supplementary fig. S4, Supplementary Material) that reside within the HFD. The *e*-values of all motifs were below 10^−5^. Asterisks above logo plots for motifs 1, 3, and 4 indicate residues that are highly conserved within the motif. Proportion of all 32 ancestral HCP-3 proteins (black) or 14 HCP-3L duplicates (blue) that have retained the motifs are shown. (*B*) *Caenorhabditis* species tree with schematics of protein motifs that are present (numbered boxes) in ancestral HCP-3 (black) or HCP-3L (blue) in each species is shown. The presence of motif 1 in *C. elegans* and motif 4 in *C. sp54* was not detected by unsupervised MAST searches but was subsequently ascertained through manual alignments (see supplementary data S4, Supplementary Material). All proteins contained a conserved, C-terminal HFD (not shown). Filled black boxes represent three motifs that show the highest retention in *Caenorhabditis* HCP-3 proteins. A structure of the *N*-terminal tail of HCP-3 in the last common ancestor of *Caenorhabditis* was inferred based on the retention and loss of motifs in the *N*-terminal tail. L1–L10 on the species tree indicate *hcp-3* duplication events as in Figure 1. A scale bar (number of residues) is shown on the bottom-right.

We took advantage of our comprehensive identification of HCP-3 and HCP-3L proteins to *de novo* identify conserved residues or motifs in their *N*-terminal tails using the MEME suite of software (Bailey et al. 2015) as previously described (Kursel and Malik 2017; see Methods). For this, we first identified motifs by analyzing all *Caenorhabditis* species encoding a single HCP-3 protein, which are more likely to have retained all motifs essential for their functions. Using this analysis, we identified 13 motifs within HCP-3 (fig. 4*A*; supplementary fig. S4, supplementary table S3, Supplementary Material), numbered sequentially from the *N*-terminus, with 11 motifs in the *N*-terminal tail and motifs 12 and 13 in the HFD. Not all 13 motifs are universally present in species encoding a single *hcp-3* gene. For example, motif 2 is present in only a subset of species examined. Based on phylogenetic analyses, we infer that motif 2 was acquired in the ancestor of a clade of eight species which includes *C. sulstoni* and *C. becei* (fig. 4*B*).

In the second step, we investigated how well these 13 motifs are conserved in species containing *hcp-3L* genes. We found that these motifs varied in their evolutionary stability and conservation. *N*-terminal tail motifs 1–11 are more variably retained than HFD motifs 12–13 (supplementary fig. S4, Supplementary Material), which are present in all HCP-3 and HCP-3L proteins as expected, except for HCP-3L3 from *C. sp54*, which has a divergent HFD. Overall, the motifs we have defined account for 48% and 63% of the total *N*-tail sequence in *C. elegans* HCP-3 and CPAR-1, respectively.

Our initial unsupervised motif analysis found that motif 3 was universally conserved in all HCP-3 and HCP-3L proteins (fig. 4*B*). In contrast, motifs 1 and 4 were universally retained in at least one paralog in each species (often both) with only a few exceptions. Recognizing that apparent “motif loss” might be the result of indels or divergence of a critical conserved residue, we manually re-examined the sequences missing either motif 1 or 4 to see if they were missed because they fell below the statistical threshold of the unsupervised motif analysis. Based on these analyses (supplementary data S4, Supplementary Material), we were able to confirm the presence of motifs 1 and 4 in all species (fig. 4*B*). Thus, three motifs (1, 3, and 4) are present in at least one HCP-3 paralog in all species. Notably, these motifs have not been identified in previous analyses of the *N*-terminal tail, highlighting the value of alignment-independent methods. These motifs include residues that are almost universally conserved in *Caenorhabditis* species (asterisks in fig. 4*A*). We predict that mutation of these residues may reveal important insight about the various functions of the HCP-3 *N*-terminal tail, including its interactions with kinetochore proteins such as KNL-2.

Motifs 5, 6, 7, 9, and 11 were less conserved, being present in 78–94% of species. For example, motif 6 appears to be lost in both HCP-3 paralogs from *C. tribulationis* and *C. afra*, while motif 11 is not found in *C. wallacei*, *C. elegans* (both paralogs), and in sister species *C. sulstoni* and *C. afra* (both paralogs). Motif 5 includes a 4-amino acid segment, ExxR (fig. 4*A*, where x represents any amino acid) that constitutes a putative cleavage motif for the separase enzyme, which initiates anaphase by cleaving the kleisin subunit of cohesin (Monen et al. 2015). Although this ExxR motif is found in both HCP-3 and CPAR-1 in *C. elegans*, only the latter is cleaved by separase (Monen et al. 2015). This suggested that the ExxR motif is necessary but not sufficient for efficient separase cleavage. Since CPAR-1 is not associated with centromeres (Gassmann et al. 2012; Monen et al. 2015), it is difficult to establish the significance of the ExxR motif, whose mutation led to no deleterious fitness consequences (Monen et al. 2015). In cases where motif 5 was missing, individual alignments of HCP-3 sequences allowed us to identify the ExxR separase motif in all HCP-3 and HCP-3L proteins, except for HCP-3L proteins from *C. latens* and *C. sp48* (supplementary fig. S5, Supplementary Material). Thus, although it is unclear whether it is required for separase cleavage or some other function, the ExxR motif is nevertheless largely conserved in all HCP-3 proteins and most HCP-3L proteins. It is possible that the cleavage site mediates the removal of the *N*-terminal tail from certain HCP-3L proteins, thereby eliminating it from a role in germline re-establishment of centromere identity (Prosée et al. 2021).

Even though motif 2 was only acquired in eight *Caenorhabditis* species (fig. 4*B*), it has been retained in at least one HCP-3 paralog of each of these species. The only instances of motif 2 loss are seen in *C. panamensis hcp-3L10* and in *C. afra hcp-3*. Our findings suggest that motif 2 is functionally important in these species despite not being universally present within all *Caenorhabditis* species. We hypothesize that some clade-specific HCP-3 protein–protein interactions or functions were acquired via motif 2 in the ancestor of these eight species.

In some instances, motif loss occurred in only one of the two HCP-3 paralogs from the same species. For example, most HCP-3L proteins lack motif 7, whereas ancestral HCP-3 in the same species usually contained this motif. Similarly, in species containing motif 5 and/or 6, the *hcp-3L* gene almost always lost these motifs, whereas the ancestral *hcp-3* maintained them. In sister species *C. brenneri* and *C. sp48*, the converse is seen, where motif 11 is maintained in the duplicate *hcp-3L* gene but lost in ancestral *hcp-3*. Overall, however, motif loss tends to occur more frequently in the *hcp-3L* paralog instead of the ancestral *hcp-3*. Thus, *hcp-3L* paralogs may be capable of performing only a subset of the functions of an ancestral *hcp-3*. This asymmetric pattern of motif loss may also explain why ancestral *hcp-3* has been universally retained in all *Caenorhabditis* species, whereas *hcp-3L* paralogs are rarely present in more than two species.

### Selective Constraints on *hcp-3* Orthologs and *hcp-3L* Paralogs

Our study represents an opportunity to evaluate the selective pressures imposed on *CenH3* genes either due to holocentricity or due to their recurrent duplication. A previous analysis had concluded there was weak evidence of positive selection from an analysis of *hcp-3* sequences from six divergent *Caenorhabditis* species whose sequence was available at that time (Zedek and Bureš 2012). However, extremely large divergence and low number of sequences can result in false signals of positive selection. Therefore, we revisited this analysis using maximum likelihood methods (see Methods). We separately analyzed *hcp-3* sequences from the two deep lineages of *Caenorhabditis* species evaluated here, as well as two subsets of species from one of the lineages for which we had enough representation (supplementary table S1*A*, Supplementary Material). In every case, we found no evidence of positive selection acting on *hcp-3* genes.

Since the presence of a paralog within the genome may affect the selective constraint on the ancestral *hcp-3* gene, we repeated the analysis by intentionally excluding all species that encode one or more *hcp-3L* paralogs (supplementary table S1*A*, Supplementary Material). Once again, we found no evidence for positive selection. Thus, in contrast to the previous study (Zedek and Bureš 2012) and in contrast to findings that *CenH3* genes from multiple other animal and plant taxa evolve under positive selection (Malik and Henikoff 2001; Talbert et al. 2004; Schueler et al. 2010; Finseth et al. 2015), we find no evidence for positive selection acting on *CenH3* genes in *Caenorhabditis*. Our inability to detect positive selection may reflect a lack of statistical power, although we note that the tree lengths used in our analysis are typical for such analyses.

Based on their presence in few species, we infer that most of the *hcp-3L* genes we identified in *Caenorhabditis* species are relatively young. Our finding that *hcp-3L* genes bore the brunt of motif loss (fig. 4) raised the possibility that many *hcp-3L* genes are not functionally constrained. To address this possibility, we carried out three types of analyses. First, we examined selective constraints acting on *hcp-3* and *cpar-1* by investigating polymorphisms within natural isolates of *C. elegans* strains that have been previously sequenced (Cook et al. 2017; supplementary fig. S6, Supplementary Material). We found only three synonymous (amino acid preserving) and zero nonsynonymous (amino acid altering) polymorphisms in *hcp-3*. In contrast, *cpar-1* contained two synonymous polymorphisms (including one commonly shared between more than 25 strains) and six nonsynonymous polymorphisms, four of which are shared among more than seven *C. elegans* strains. Some of these polymorphisms arise in otherwise conserved positions in the *N*-terminal tail (fig. 4; supplementary fig. S6, Supplementary Material) or HFD, implying that they are likely deleterious for function. In addition to nonsynonymous changes, we found at least two strains that may have disrupted *cpar-1*, via either a frameshift or a splice site mutation. Based on this comparison, we infer that *cpar-1* is evolving under lower functional constraints than *hcp-3* in *C. elegans*.

Second, we tested whether *hcp-3L* paralogs are generally evolving under fewer stringent functional constraints than *hcp-3* genes. For this, we calculated dN/dS values, which measure the ratio of the normalized rates of nonsynonymous substitutions to synonymous substitutions. A lower dN/dS ratio is reflective of higher functional constraints, whereas a dN/dS ratio of close to 1 is reflective of lack of functional constraints for protein-coding function. We calculated dN/dS values in pairwise comparisons of the HFD of *hcp-3L* orthologs present in two distinct species: *hcp-3L4* in *C. latens* and *C. remanei*, *hcp-3L2* in *C. sinica* and *C. tribulationis*, and *hcp-3L5* in *C. brenneri* and *C. sp48* (supplementary table S1*B*, Supplementary Material). We obtained dN/dS ratios of 0.02, 0.04, and 0.08, respectively. These values are considerably lower than 1, suggesting that all three paralogs have been retained under functional constraint for protein-coding function during the divergence of the respective *Caenorhabditis* species. Moreover, in all three cases, we found that dN/dS values for *hcp-3L* orthologs were comparable to or lower than corresponding *hcp-3* orthologs from the same species (supplementary table S1*B*, Supplementary Material). For comparison, the dN/dS values for pairwise comparisons of ancestral *hcp-3* from *C. latens*/*C. remanei*, *C. sinica*/*C. tribulationis*, and *C. brenneri*/*C. sp48* are 0.18, 0.02, and 0.03, respectively. Thus, unlike *cpar-1* in *C. elegans*, we find that *hcp-3L* paralogs have evolved under similar or even more stringent constraints than ancestral *hcp-3* genes at least in some *Caenorhabditis* species.

Given this finding, we revisited the age of the *hcp-3L* paralogs in *Caenorhabditis* species in a third analysis. Unlike dN or dN/dS values, dS values are relatively unaffected by selective constraints and provide a more reliable proxy for their divergence from *hcp-3* ancestors. We calculated the synonymous divergence (dS) between the HFD of *hcp-3L* paralogs whose closest relatives are *hcp-3* orthologs from the same species (fig. 2). These dS values range from 0.15 (for *C. afra*) to 0.74 (for *C. doughertyi*) (supplementary table S1*B*, Supplementary Material). These dS values are considerably lower than seen between *Drosophila CenH3* paralogs in the same species (e.g., *D. virilis*). Although we lack reliable molecular clock-like estimates to convert these dS values to millions of years of divergence (Cutter 2008), the dS values are high enough to imply that a majority of these *hcp-3L* paralogs have been functionally retained for several million years, even though most of them have not been retained across multiple speciation events (fig. 1).

**Fig. 5.**
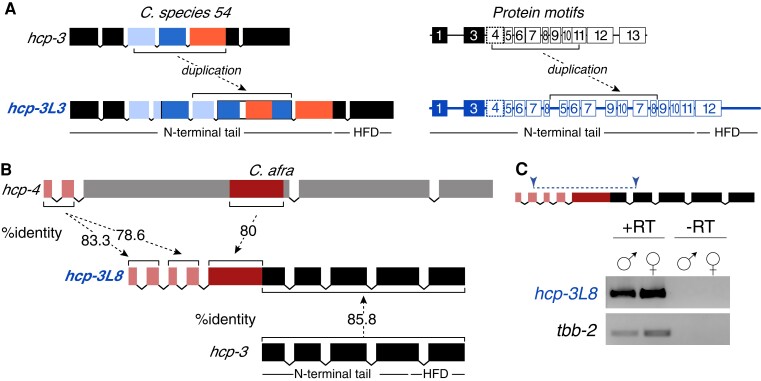
Two unusual *Caenorhabditis hcp-3L* paralogs arose by internal duplication or gene fusion. (*A*) Schematic of the exon structure (left) and protein motif structure (right) of *C. sp54 hcp-3* (top) and *hcp-3L3* (bottom). Portions of *hcp-3* exon 3 (light blue), exon 4 (dark blue), and exon 5 (orange) are duplicated within the *N*-terminal tail of *hcp-3L3* (dashed arrow). Similarly, motifs 5–10 are duplicated within the *N*-terminal tail of HCP-3L3. Motif 13 resides within the HFD and is missing in HCP-3L3. The HFD is not within the duplicated region. (*B*) Schematic of the exon structure of *C. afra hcp-3L8* (middle) with homology to *C. afra hcp-4* (top) and *C. afra hcp-3* (bottom). The first five exons of *hcp-3L8* are homologous to *C. afra hcp-4* exons 1 and 2 (light red) as well as a portion of exon 3 (dark red). The last five exons of *hcp-3L8* are homologous to *C. afra hcp-3* (black). The HFD and the *N*-terminal tail of *hcp-3* are denoted. Percent amino acid identity between protein-coding exons are shown. (*C*) Primers designed to span exons that are homologous to *hcp-3* and *hcp-4* within *hcp-3L8* (top). Schematic of the gene shows primers used to amplify the *hcp-4-hcp-3* fusion region (top, blue) in RT-PCR of *C. afra hcp-3L8* and *tbb-2* in males and females (bottom) to confirm expression of a chimeric transcript. +RT and −RT indicate cDNA preparation with or without reverse transcriptase enzyme, respectively.

The overall selective pressure acting on *hcp-3L* paralogs is that of purifying selection or evolutionary constraint. However, our comparison of HFD between *hcp-3* and *hcp-3L3* from *C. sp54* revealed a dN/dS of 1.74 in a maximum likelihood test, although this is not statistically significantly different from the neutral expectation of dN/dS = 1. Based on the phylogeny of *CenH3* HFD (fig. 2), we could infer that *C. sp44 hcp-3* is an outgroup to the two *C. sp54 hcp-3* genes. We compared *C. sp44 hcp-3* to either *hcp-3* or *hcp-3L3* from *C. sp54*. These analyses revealed a lower dN/dS in a comparison between the two ancestral *hcp-3* orthologs (dN/dS = 0.09) than between *C. sp44 hcp-3* and *C. sp54 hcp-3L3* (dN/dS = 0.34). This implies that it is the unusual paralog, *hcp-3L3*, that has evolved more rapidly. This combined with our finding that HCP-3L3 contains duplications of the *N*-terminal tail motifs (fig. 5*A*) suggests the possibility of incipient neofunctionalization of the *hcp-3L3* paralog in *C. sp54*.

### Duplication of Other Centromere-Localized Proteins in *Caenorhabditis* Species

In most cases, the protein sequence of HCP-3 paralogs can be confidently aligned to the ancestral HCP-3, indicating clear homology. However, aligning *C. afra* HCP-3 and *C. afra* HCP-3L8 revealed that the paralog contained an additional 198 amino acids on its *N*-terminus. This region was not homologous to HCP-3. To our surprise, we found that this segment was instead homologous to CENP-C (known as HCP-4 in *C. elegans*). HCP-4 and HCP-3 directly interact with each other in *C. elegans* (Oegema et al. 2001) and in other eukaryotes. *C. afra hcp-3L8* contained two copies of *C. afra hcp-4* exons 1 and 2, followed by a partial copy of *hcp-4* exon 3. These *hcp-4* homologous segments are contiguous with *hcp-3*-homologous sequence to constitute the *hcp-3L8* coding sequence (fig. 5*B*). We used RT-PCR to confirm that *hcp-3L8* was transcribed as a single transcript containing homology to both *hcp-4* and *hcp-3* sequences (fig. 5*C*). Therefore, *C. afra hcp-3L8* is a chimera of *hcp-4* and *hcp-3*. In addition to this *hcp-4-hcp-3* fusion gene, *C. afra* also maintains its ancestral *hcp-3* and *hcp-4* genes. The functional roles of the HCP-4-like regions present within *hcp-3L8* are unknown. However, a conserved CENP-C motif is absent in this chimera. The conserved CENP-C motif, which mediates the interaction with the CenH3 nucleosome (Kato et al. 2013), is present at the C-terminus of *C. elegans* HCP-4 (Moore and Roth 2001). Thus, loss of the CENP-C motif in HCP-3L8 is not unexpected since the HCP-4 and HCP-3 segments are already physically linked to each other in this chimeric protein.

**Fig. 6.**
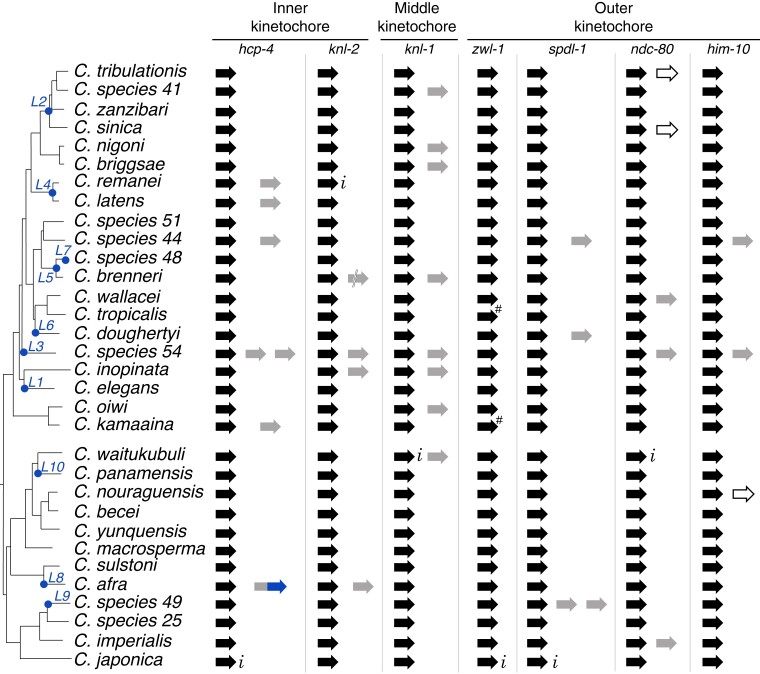
Duplication of kinetochore proteins in *Caenorhabditis* species. A schematic representation of ancestral (black) and duplicate (gray) copies of seven kinetochore genes (*hcp-4*, *knl-2*, *knl-1*, *zwl-1*, *spdl-1*, *ndc-80*, and *him-10*) shown alongside a *Caenorhabditis* species tree. *hcp-3* duplication events are denoted as a blue dot on the species tree, as in Figure 1. The unique fusion between *C. afra hcp-4* and *hcp-3* duplicates is shown in gray and blue. Incomplete sequence information in genomic scaffolds is denoted with *i* and apparent pseudogenes are denoted as unfilled arrows. Double slash in *C. brenneri knl-2* duplicate indicates the sequence was split between two scaffolds. # indicates two potential pseudogenization events in *zwl-1* that are likely to represent sequencing errors.

Encouraged by this finding of *hcp-4* duplication and fusion with *hcp-3* in *C. afra*, we investigated whether other centromere-localized proteins have also duplicated and diversified like *hcp-3*. We performed similar paralog searches for proteins from the inner kinetochore (*hcp-4* and *knl-2*), middle kinetochore (*knl-1*), and outer kinetochore (*him-10*, *ndc-80*, *spdl-1*, and *zwl-1*). We found an intact copy of each ancestral gene in every species (fig. 6) except for two instances where we were unable to identify full-length intact *zwl-1* genes (in *C. kamaaina* and *C. tropicalis*) (“#” in fig. 6). We found instances of duplications for all kinetochore proteins except *zwl-1*. These duplications either appear to be retained with an intact open reading frame (filled, gray arrows), or are interrupted (double lines), or show clear signs of pseudogenization (unfilled arrows) (fig. 6). In the 32 species examined, we found seven *hcp-4* duplicates, four *knl-2* duplicates (including a pseudogene in *C. brenneri*), eight *knl-1* duplicates, four *spdl-1* duplicates, five *ndc-80* duplicates (including two pseudogenes), and three *him-10* duplicates (including one pseudogene). Duplications of inner and middle kinetochore proteins were only marginally more prevalent than duplications of outer kinetochore proteins. Interestingly, we observed several instances of partial intron losses that occurred recurrently in genes encoding ancestral and paralog outer kinetochore proteins (supplementary fig. S7, Supplementary Material) like what we observed previously for *hcp-3* and *hcp-3L* genes (supplementary fig. S1, Supplementary Material). Overall, our analyses suggest that in addition to HCP-3, other kinetochore proteins are also undergoing duplication and diversification in *Caenorhabditis* species.

To understand the evolutionary constraints on *Caenorhabditis* kinetochore proteins, we analyzed these genes using maximum likelihood methods. We found no evidence of positive selection acting on ancestral *hcp-4*, *knl-1*, *knl-2*, *zwl-1*, *spdl-1*, *ndc-80*, and *him-10* genes in either the *C. elegans* or *C. afra* clades (supplementary table S1*C*, Supplementary Material). Next, we examined the evolutionary constraints acting on paralogs of kinetochore proteins by comparing the paralogs to the ancestral kinetochore genes from the same species. In all cases except two, we found strong evidence that the duplicates are retained under strong purifying selection (supplementary table S1*D*, Supplementary Material). For *knl-2* in *C. inopinata* and *ndc-80* in *C. sp54*, we could not rule out the null hypothesis of neutral evolution.

We investigated whether any kinetochore protein paralogs have been co-retained with *hcp-3L* paralogs, which would suggest a concerted duplication and retention of multiple kinetochore proteins, consistent with significant specialization. We found that four of six independent *hcp-4* duplications coincided with retention of *hcp-3L* paralogs in the same species (fig. 6). These include an *hcp-4* paralog whose origin coincides with the *hcp-3L4* paralog in *C. latens* and *C. remanei*, two *hcp-4* paralogs that co-occur with *hcp-3L3* in *C. sp54*, and the *hcp-4-hcp-3* fusion gene in *C. afra* (*hcp-3L8*). Thus, 4 of 14 species containing an *hcp-3L* paralog also encode a (complete or partial) *hcp-4* paralog, whereas 2 of 18 species lacking *hcp-3L* paralogs encode a *hcp-4* paralog: *C. sp44* and *C. kamaaina*. Thus, there is no statistically significant evidence of co-retention (*P* = 0.36), indicating that the duplication or retention of *hcp-3* and *hcp-4* paralogs may be independent.

Other kinetochore proteins analyzed also largely reflect this pattern of independent duplication. Even though KNL-2 is required to deposit HCP-3 proteins at centromeres in *Caenorhabditis* species (Maddox et al. 2007; de Groot et al. 2021; Prosée et al. 2021), there does not appear to be a significant pattern of co-retention with *hcp-3L* paralogs. The one exceptional species is *C. sp54*, which encodes an *hcp-3L3* paralog, two *hcp-4* paralogs, a *knl-2* paralog, a *knl-1* paralog, an *ndc-80* paralog, and a *him-10* paralog. If the proteins encoded by these paralogs exclusively interact with each other, this species may represent an intriguing case of incipient kinetochore specialization.

## Discussion

Our analyses reveal that *hcp-3* has duplicated at least ten independent times within *Caenorhabditis* species. In contrast to ancient co-retention of *CenH3* paralogs in plants, *Drosophila*, mosquito species (Maheshwari et al. 2015; Kursel and Malik 2017; Kursel et al. 2020, 2021), and even holocentric *Meloidogyne* nematode species (Despot-Slade et al. 2021), we observed only a few cases of *hcp-3L* paralogs that are shared across two or three *Caenorhabditis* sister species, although this may partly reflect density of species sampling in these different taxonomic groups. Our findings suggest that most of the *hcp-3L* paralogs we have found are relatively young, assuming that the relative ages of *Caenorhabditis* and *Drosophila* species analyzed are comparable (Cutter 2008).

Our comprehensive phylogenomic approach in *Caenorhabditis* nematodes uncovered two novel aspects of CenH3 evolution. First, we uncovered a detailed molecular architecture of the *N*-terminal tail of HCP-3 proteins (fig. 4). The HCP-3 *N*-terminal tail is dispensable for mitotic chromosome segregation and centromere maintenance during *C. elegans* development (Prosée et al. 2021) but is essential in establishing a functional HCP-3 distribution in the germline, which is maintained in the subsequent generation throughout development. At least part of this functionality of the HCP-3 *N*-terminal tail stems from its interactions with kinetochore proteins like KNL-2 (de Groot et al. 2021; Prosée et al. 2021). Thus far, however, the molecular architecture of the interactions of HCP-3 with other kinetochore proteins like KNL-2 has been only crudely defined. Like in other eukaryotic lineages, the *N*-terminal tail of HCP-3 proteins is much more divergent than the HFD. Thus, comparisons of functional domains in CenH3 *N*-terminal tails between taxonomic groups or even within *Caenorhabditis* are very difficult, exacerbating the difficulty in defining functional domains within HCP-3’s *N*-terminal tail. Our description of 11 motifs in HCP-3 *N*-terminal tails, including three that are nearly universally conserved, provides an important resource for the fine-scale dissection of the various protein–protein interactions mediated by the *N*-terminal tail and the functional role these interactions play in centromere biology. In particular, the three conserved motifs contain residues that are as well conserved as many HFD residues across *Caenorhabditis* species.

We propose that these *N*-terminal tail motifs are sites of previously proposed or novel protein–protein interactions, either with kinetochore proteins or with other chromatin factors that could intersect with holocentromere formation or maintenance. Consequently, motif gains or losses could indicate gains or losses of HCP-3 interactions with partner proteins. We observe one unambiguous case of motif gain in one clade of *Caenorhabditis* species. Motif 2 likely represents a novel protein–protein interaction module important for *CenH3* function at least in those species. We also observe several cases of motif degeneration or loss. Unlike in *Drosophila CenH3* paralogs (Kursel and Malik 2017), we see little evidence for motif redistribution between the paralog and ancestral *hcp-3* genes, which would suggest subfunctionalization; the only exception is motif 2 that appears to be present in either HCP-3 or HCP-3L proteins, but not both. Overall, we find that motif loss or degeneration preferentially occurs in *hcp-3L* paralogs rather than ancestral *hcp-3*, suggesting that the paralogs progressively lose ancestral functions and interactions. Since tail-less HCP-3 proteins can still function in mitosis (Prosée et al. 2021), it is tempting to speculate that HCP-3L paralogs could still function in mitosis despite progressive loss of *N*-terminal motifs.

The remarkable example of a chimeric gene in *C. afra*, where an HCP-3L protein is fused to an inner kinetochore protein, HCP-4 (CENP-C in mammals; fig. 5) exemplifies an instance where previously conserved motifs could be lost. HCP-3 and HCP-4 physically interact in many eukaryotes to form the kinetochore complex during mitosis. The fusion of these two proteins in HCP-3L8 guarantees a protein–protein interaction, which is consistent with the loss of the CENP-C motif (Kato et al. 2013) required for HCP-3L and HCP-4 interactions. This could also lead to loss of HCP-3L *N*-terminal tail motifs required for HCP-4 association.

The second major conclusion from our evolutionary analyses is the unusually rapid cadence of turnover of *hcp-3* paralogs in *Caenorhabditis* species. Nearly half of the species we analyzed contain an *hcp-3* paralog. Yet, in contrast to analyses in *Drosophila* and mosquito lineages, where duplicates were older and fewer in number (Kursel and Malik 2017; Kursel et al. 2020), the *Caenorhabditis* paralogs were acquired through ten independent duplication events. Most paralogs have only been retained in a single species with only one *hcp-3L* paralog being present in more than two species. Previous analyses suggest that *C. elegans* have a higher gene duplication rate than other species including *D. melanogaster* (Lynch and Conery 2000; Pan and Zhang 2007; Lipinski et al. 2011), potentially as high a duplication rate per gene as 0.02 every million years (Lynch and Conery 2000). This high rate of gene duplication may account for the higher number of *hcp-3* duplications we observe in *Caenorhabditis*. However, these analyses also suggest that the vast majority of gene duplications that arise in *C. elegans* are efficiently purged by natural selection (Lipinski et al. 2011). In contrast, our findings suggest that many *hcp-3L* paralogs are retained under purifying selection for significant periods of time.

Our evolutionary analyses thus reveal an unusual “revolving-door” of *hcp-3L* paralogs in *Caenorhabditis* species. Under this regime, gene duplication is frequent, *hcp-3L* paralogs are retained under purifying selection for a significant evolutionary period before eventually either degenerating (e.g., possibly *cpar-1* in *C. elegans*) or being lost entirely (e.g., possibly *hcp-3L2* in *C. zanzibari*), returning to the ancestral state of the genome encoding only a single *hcp-3* gene. This cadence is unprecedented among most other taxonomic groups where *CenH3* duplications have been investigated. Even the high number of *hcp-3* duplications we have observed is likely an under-estimate of the true number, since extant species represent only one evolutionary snapshot. Indeed, our study implies that many previously arising *hcp-3L* paralogs have been lost or degenerated beyond recognition during *Caenorhabditis* evolution. This is akin to the “revolving door” of HP1-family proteins previously reported in *Drosophila* (Levine et al. 2012). Although we have not evaluated all of them in the same level of detail, duplications of other kinetochore proteins in *Caenorhabditis* also appear to occur with a similar revolving-door dynamic.

What could account for this revolving-door, that is, the short-term evolutionary retention of *hcp-3L* paralogs and their long-term loss or degeneration? We consider several possibilities for the sources of transient selective pressure to retain *CenH3* paralogs. First, this pattern could result from specialization of kinetochore paralogs for functions that are unrelated to chromosome segregation, as has been recently shown in *Caenorhabditis* and *Drosophila* neurodevelopment (Cheerambathur et al. 2019; Zhao et al. 2019). A previous study showed that CPAR-1 localizes to chromosomes but not centromeres in *C. elegans* (Monen et al. 2015), although it is unclear whether this is typical for other HCP-3L paralogs.

A second explanation for this pattern might be subfunctionalization of *CenH3* paralogs for tissue- or sex-specific or meiosis-specific functions, as is proposed in *Drosophila* species (Despot-Slade et al. 2021; Kursel et al. 2021). Unlike monocentric chromosomes, holocentric chromosomes experience inherent challenges during meiosis, which have been overcome in different taxa via different means (Melters et al. 2012; Marques and Pedrosa-Harand 2016). A recent study in *Meloidogyne* nematode species found that an ancestral αCenH3 is deeply conserved for function in mitosis whereas more rapidly evolving CenH3 paralogs lost mitotic function (Despot-Slade et al. 2021). However, we found no evidence of sex-specific expression of *CenH3* paralogs in *Caenorhabditis* species. Moreover, unlike in most eukaryotes, *C. elegans* chromosomes connect to the meiotic spindle by a CenH3-independent mechanism (Monen et al. 2005). Therefore, at least in *C. elegans*, *hcp-3* is entirely dispensable for meiotic chromosome segregation (Monen et al. 2005). This relaxes constraints to maintain meiotic functions on *hcp-3* genes but cannot explain the revolving-door pattern.

A third possible explanation for the transient retention of *hcp-3* paralogs is suppression of either “centromere-drive” or “holokinetic drive”. Currently, it is unclear whether centromere drive could occur in holocentric organisms (Zedek and Bureš 2012, 2016; Krátká et al. 2021). Although a previous study reported weak evidence of positive selection using an analysis of *hcp-3* from six highly diverged *Caenorhabditis* species (Zedek and Bureš 2012), our comprehensive reanalysis of *hcp-3* evolution across a much more densely sampled series of closely related species revealed no evidence of positive selection (supplementary table S1*B*, Supplementary Material). Similarly, the *αCenH3* gene required for mitosis is deeply conserved and slowly evolving in *Meloidogyne* nematodes although other *CenH3* paralogs appear to be rapidly evolving (Despot-Slade et al. 2021). Asymmetric meiosis in nematode species could also lead to another form of drive, leading to preferential inheritance of larger or smaller holocentric chromosomes (“holokinetic drive”), which could explain the observed negative correlation between chromosome number and genome size in many holocentric lineages (Bureš and Zedek 2014). If either of these drive mechanisms occur in *Caenorhabditis* species, then *hcp-3L* paralogs could arise and be temporarily retained as drive-suppressors, but only while the driving elements were still present in the genome. This suppression might result in loss of these driving elements from the genome, rendering *hcp-3L* gene functions superfluous and resulting in subsequent loss of these paralogs. Given the uncertainty about the existence of centromere-drive or holokinetic drive in nematodes, or the role that *hcp-3L* paralogs might play in either process, we cannot elaborate further on this possibility.

We favor a fourth hypothesis, in which the holocentricity of *Caenorhabditis* species, with HCP-3 distributed along the length of the chromosomes, might itself lead to the revolving-door dynamics of centromeric proteins. CenH3 incorporation into nucleosomes at holocentromeres is more plastic than at monocentromeres. Since CenH3 does not have to associate with specific sequences or chromosomal regions, holocentric chromosomes more easily tolerate chromosome breakage, fusion, or rearrangements. Indeed, even prior to clear cytological evidence, holocentric organisms were observed to maintain fertility despite radiation-induced chromosome breaks (Schrader 1935; Melters et al. 2012). Moreover, even completely foreign DNA can form mini-chromosomes that assemble centromeres and be stably propagated (Zhu et al. 2018; Lin and Yuen 2020; Lin et al. 2021). Nevertheless, centromere distribution in holocentric organisms is not random. Although HCP-3 presence is partially linked to certain “HOT (High Occupancy Target) sites” in *C. elegans* (Steiner and Henikoff 2014), the overall pattern of centromere establishment in *C. elegans* appears to be predominantly linked to transcriptionally repressed genomic regions in the germline. This pattern of centromere definition via transcriptional inactivity is seen in both *C. elegans* and in the CenH3-devoid *Bombyx mori* (Gassmann et al. 2012; Steiner and Henikoff 2014; Senaratne et al. 2021). In contrast, some holocentric species like *Meloidogyne* nematodes and *Rhynchospora* plants localize their CenH3 proteins to specific repeats found distributed over the genome (Marques et al. 2015; Despot-Slade et al. 2021; Hofstatter et al. 2022).

Although a transcriptional quiescence-dependent mode of centromere definition is more tolerant of genomic rearrangements than monocentric organisms, it could also be subject to transient stress. This stress could be imposed by either chromosomal rearrangements or transposon invasion, which can quickly and dramatically alter the landscape of transcription and repression in the germline. In such circumstances, it might be advantageous to retain HCP-3L paralogs to temporarily increase the dosage of proteins required to correctly establish centromere identity, as has been proposed in some plant lineages (Evtushenko et al. 2021). Alternatively, it may be advantageous to express HCP-3 proteins with slightly altered sequences and localization preferences, allowing restoration of optimal centromere distributions even after periods of such “genomic stress”. Under either scenario, eventual amelioration of the genomic stressor (e.g., decay or silencing of the invading transposable element) would render *hcp-3L* paralogs superfluous and these would be lost. Therefore, we hypothesize that holocentric species like *C. elegans*, which rely on a transcriptional quiescence-dependent mode of centromere definition, may be prone to revolving-door dynamics of their kinetochore proteins.

Different *Caenorhabditis* species might represent different stages of the revolving-door process for kinetochore proteins. Species like *C. sp54*, which possess paralogs of five of seven kinetochore genes investigated, may be actively selecting for the retention and function of these paralogs. In contrast, species like *C. elegans*, with a possibly nonessential *cpar-1* and no other kinetochore paralogs, may have already overcome the need for such innovation. We, therefore, predict that functional consequences of kinetochore paralog loss in different *Caenorhabditis* species will differ based on their stage of genetic innovation. Our study underlines the need for the analysis of nonmodel organisms and the value of evolutionary comparisons to reveal novelties even in well-studied cellular pathways.

## Methods

### Strain Maintenance

All strains were cultured on Nematode Growth Medium (NGM) plates seeded with 200 μl OP50 at 20 °C using standard methods (Brenner 1974).

### Strains Used

**Table:** 

N2	*C. elegans*
DF5081	*C. japonica*
JU727	*C. sinica*
JU1333	*C. doughertyi*
JU2744	*C. tribulationis*
JU1199	*C. afra*
VX88	*C. latens*
QG702	*C. panamensis*

### Identification of *hcp-3* and Kinetochore Protein Homologs in Sequenced Genomes

To identify *hcp-3* paralogs and orthologs, we iteratively queried the assembled genomes of 32 *Caenorhabditis* species: *C. tribulationis*, *C. sp41*, *C. zanzibari*, *C. sinica*, *C. nigoni*, *C. briggsae*, *C. remanei*, *C. latens, C. sp51*, *C. sp44*, *C. sp48*, *C. brenneri*, *C. wallacei, C. tropicalis*, *C. doughteryi*, *C. sp54*, *C. inopinata*, *C. elegans, C. oiwi*, *C. kamaaina*, *C. waitukubuli*, *C. panamensis*, *C. nouraguensis*, *C. becei*, *C. yunquensis*, *C. macrosperma*, *C. sulstoni*, *C. afra, C. sp49*, *C. sp25*, *C. imperialis*, and *C. japonica* (supplementary data S2, Supplementary Material). We used tBLASTn (Altschul et al. 1990, 1997) on each species’ genome (Stevens et al. 2019) to perform a homology-based search starting with *C. elegans* HCP-3 (WBGene00001831) as our query. We used a combination of gene predictions, publicly available RNA sequencing data, *hcp-3* alignments, and splice site predictions to annotate intron–exon regions of all *hcp-3* genes that were found. To ensure that we had not missed any *hcp-3* paralogs, we repeated our analyses querying each species’ hits on their own genome using tBLASTn and did not retrieve additional hits. To identify paralogs and orthologs of kinetochore proteins (fig. 6), we repeated this same homology-search procedure starting with *C. elegans* HCP-4 (WBGene00001832), KNL-1 (WBGene00002231), KNL-2 (WBGene00019432), ZWL-1 (WBGene00021460), SPDL-1 (WBGene00015515), NDC-80 (WBGene00003576), and HIM-10 (WBGene00001869). We used http://blast.caenorhabditis.org/ to perform all tBLASTn analyses using pre-set parameters and setting an *e*-value threshold of at least 10^−1^ to obtain all possible paralogs.

Synteny was used to determine *hcp-3* orthology across *Caenorhabditis* species. We identified annotated genes immediately upstream and downstream of *hcp-3* and *hcp-3L* genes. We then used these neighboring genes as queries for tBLASTn searches of the *C. elegans* genome to identify the orthologous syntenic genes (fig. 1). Dissimilar flanking genes for different *hcp-3L* paralogs provide support for the phylogenetic inference that they were acquired through independent *hcp-3* duplication events. In some cases, *hcp-3 or hcp-3L* genes were found in small genomic scaffolds or at the end of scaffolds, reducing our ability to identify upstream or downstream syntenic genes. In the latter case, we analyzed additional genes in the direction (upstream or downstream) that had sufficient genomic information available on the same scaffold. The absence of *hcp-3* in the ancestral locus in *C. sp49 hcp-3* and of *hcp-3L2* in the duplicate locus in *C. zanzibari* was confirmed by using tBLASTn of each gene in the expected locus, resulting in no detectable homologous gene sequence.

### Phylogenetic Analyses

All protein alignments were performed using the MUSCLE algorithm (Edgar 2004) in Geneious Prime 2019.2.3 (https://www.geneious.com). Codon-based nucleotide alignments were created using the MUSCLE (codon) feature in MEGAX (Kumar et al. 2018). We used only the HFD for phylogenetic inference and used the maximum likelihood method implemented in MEGA11 (Stecher et al. 2020; Tamura et al. 2021). Our amino acid-based phylogeny used the JTT model (Jones et al. 1992) and our nucleotide-based phylogeny used the General Time Reversible model (Nei and Kumar 2000). We inferred the bootstrap consensus tree from 100 replicates. Initial tree(s) for the heuristic search were obtained automatically by applying Neighbor-Joining and BioNJ algorithms to a matrix of pairwise distances estimated using the Maximum Composite Likelihood (MCL) approach, and then selecting the topology with superior log likelihood value. A discrete Gamma distribution was used to model evolutionary rate differences among sites (five categories (+*G*, parameter = 0.8943)). The rate variation model allowed for some sites to be evolutionarily invariable ([+*I*], 26.05% sites). All positions with less than 95% site coverage were eliminated, that is, fewer than 5% alignment gaps, missing data, and ambiguous bases were allowed at any position (partial deletion option). There was a total of 267 nucleotide positions in the final dataset between all HCP-3 and HCP-3L HFD amino acid sequences. Supplementary table S4, Supplementary Material presents the pairwise distances and number of differences, respectively, between all *hcp-3* and *hcp-3L* HFD coding sequences.

### Motif Analyses

Thirteen motifs were identified using MEME (Bailey et al. 2015) on predicted, full-length HCP-3 protein sequences from species lacking *hcp-3* paralogs (*C. zanzibari*, *C. nigoni*, *C. briggsae*, *C. sp51*, *C. sp44*, *C. wallacei*, *C. tropicalis*, *C. inopinata*, *C. oiwi*, *C. kamaaina*, *C. waitukubuli*, *C. nouraguensis*, *C. becei*, *C. yunquensis*, *C. macrosperma*, *C. sulstoni*, *C. sp25*, *C. imperialis*, and *C. japonica*). The *e*-values of all 13 discovered motifs were below 10^−5^. Motif logo plots were generated and downloaded from MEME. The presence or absence of these motifs in all HCP-3 and HCP-3L proteins was determined by using MAST (Bailey and Gribskov 1998). We considered a motif as present in a protein by using default parameters in MAST and a *P*-value below 10^−4^.

Since the *N*-terminal tails of HCP-3 and its paralogs are highly divergent, we were not able to identify the separase motif efficiently via motif analyses. To identify the presence of the ExxR separase motif, we separately aligned each HCP-3 or HCP-3L protein sequence with *C. elegans* HCP-3 and CPAR-1 (HCP-3L1) either individually or together. This alignment was used to generate the predicted separase motifs shown in supplementary fig. S5, Supplementary Material.

### Analysis of Evolutionary Selective Pressures

To analyze selective pressures on *CenH3* genes, we compared rates of synonymous (dS) to nonsynonymous (dN) substitutions among *hcp-3* and *hcp-3L* genes. dN and dS between all pairwise combinations of *CenH3* genes were determined using SNAP (Korber 2000; www.hiv.lanl.gov) on a codon alignment of the HFD (supplementary table S1*A*, Supplementary Material). dN/dS ratios were used to determine the selective pressures acting on *CenH3* genes.

For all other tests, we generated codon alignments using MUSCLE (Edgar 2004), and manually adjusted them to improve alignments if needed. We also trimmed sequences to remove alignment gaps and segments of the sequence that were unique to only one species. We found no evidence of recombination for any of these alignments using the GARD algorithm at datamonkey.org (Kosakovsky Pond et al. 2006). We used the alignment to generate a tree using PhyML maximum-likelihood methods with the HKY85 substitution model (Guindon et al. 2010).

We analyzed selective pressures on *Caenorhabditis hcp-3* and kinetochore proteins using the codeml algorithm from the PAML suite (Yang 1997; supplementary table S1*A*, Supplementary Material online). We generated codon alignments using MUSCLE (Edgar 2004) via Geneious’s Translation Align tool which we manually adjusted if needed to improve alignments. These alignments were used to generate trees using PhyML maximum-likelihood methods with the HKY85 substitution model (Guindon et al. 2010). To test whether any residues evolve under positive selection, we compared likelihoods between model 8 (where there are 10 classes of codons with dN/dS between 0 and 1, and a 11th class with dN/dS > 1) and model 7 (which disallows codons with dN/dS > 1) or model 8a (where the 11th class has dN/dS fixed at 1). To test whether duplicates were evolving under positive of purifying selection, we compared the likelihood of model 0 with dN/dS fixed at 1 (neutral) with that of model 0 with dN/dS estimated from the alignment. In both cases, to determine statistical significance, we performed likelihood-ratio tests between the two models to a χ^2^ distribution with the degrees of freedom reflecting the difference in the number of parameters between the models being compared (Yang 1997).

### *C. elegans* HCP-3 and CPAR-1 Polymorphisms

To determine natural variation in *C. elegans hcp-3* and *cpar-1* genes (supplementary fig. S6, Supplementary Material), we used the *Caenorhabditis elegans* Natural Diversity Resource (Cook et al. 2017). The synonymous mutations in *hcp-3*, as well as the frameshift, synonymous, and nonsynonymous mutations in *cpar-1* were identified by the CeNDR variant annotation feature. The *cpar-1* partial deletion was found manually by looking at whole-genome sequencing reads from *C. elegans* strain ECA740 mapped onto the N2 reference genome.

### RT-PCR

Total RNA was isolated using TRIzol (Fisher Scientific) from 50 to 100 L4 or young adult males, females, or hermaphrodites or from a near starved plate of mixed-stage animals. RNA was extracted by chloroform extraction, precipitated using isopropanol, washed with ethanol, and resuspended in 20 µl of nuclease-free water. Next, RNA was treated with DNase I (New England Biolabs, 2 units/µl) at 37°C for 60 min followed by heat inactivation at 75°C for 10 min. DNase-treated RNA was purified using the RNA Clean and Concentrator-5 kit (Zymo Research) and converted to cDNA using SuperScript III Reverse Transcriptase (Invitrogen) using polydT primers as per manufacturer’s recommendations. RNA concentrations used to make cDNA were not kept the same between whole plate, male, and female/hermaphrodite samples except for samples from *C. afra* (in fig. 5*C*), *C. remanei* and *C. sinica*. PCR was done on cDNA using Phusion High-Fidelity DNA Polymerase Kit (New England Biolabs) guidelines according to the manufacturer’s recommendations using primers for *hcp-3*, *hcp-3L*, and *tbb-2*. All primer sequences used are listed in supplementary table S2, Supplementary Material.

## Supplementary Material

msac206_Supplementary_Data

## Data Availability

The data underlying this article are available in the article and in its online supplementary material. Supplementary data files include sequence alignments used to generate phylogenetic trees and for evolutionary analyses and sequences of all HCP-3 and kinetochore protein orthologs and paralogs described in the paper.
