# Supplementary material for: Recurrent but Short-Lived Duplications of Centromeric Proteins in Holocentric Caenorhabditis Species
Source: Mol Biol Evol. 2022 Sep 29;39(10):msac206. doi: 10.1093/molbev/msac206 (PMC9577544; doi:10.1093/molbev/msac206)
Supplement: msac206_Supplementary_Data [file msac206_supplementary_data.zip › SupplFigsS1-S7combined.pdf]

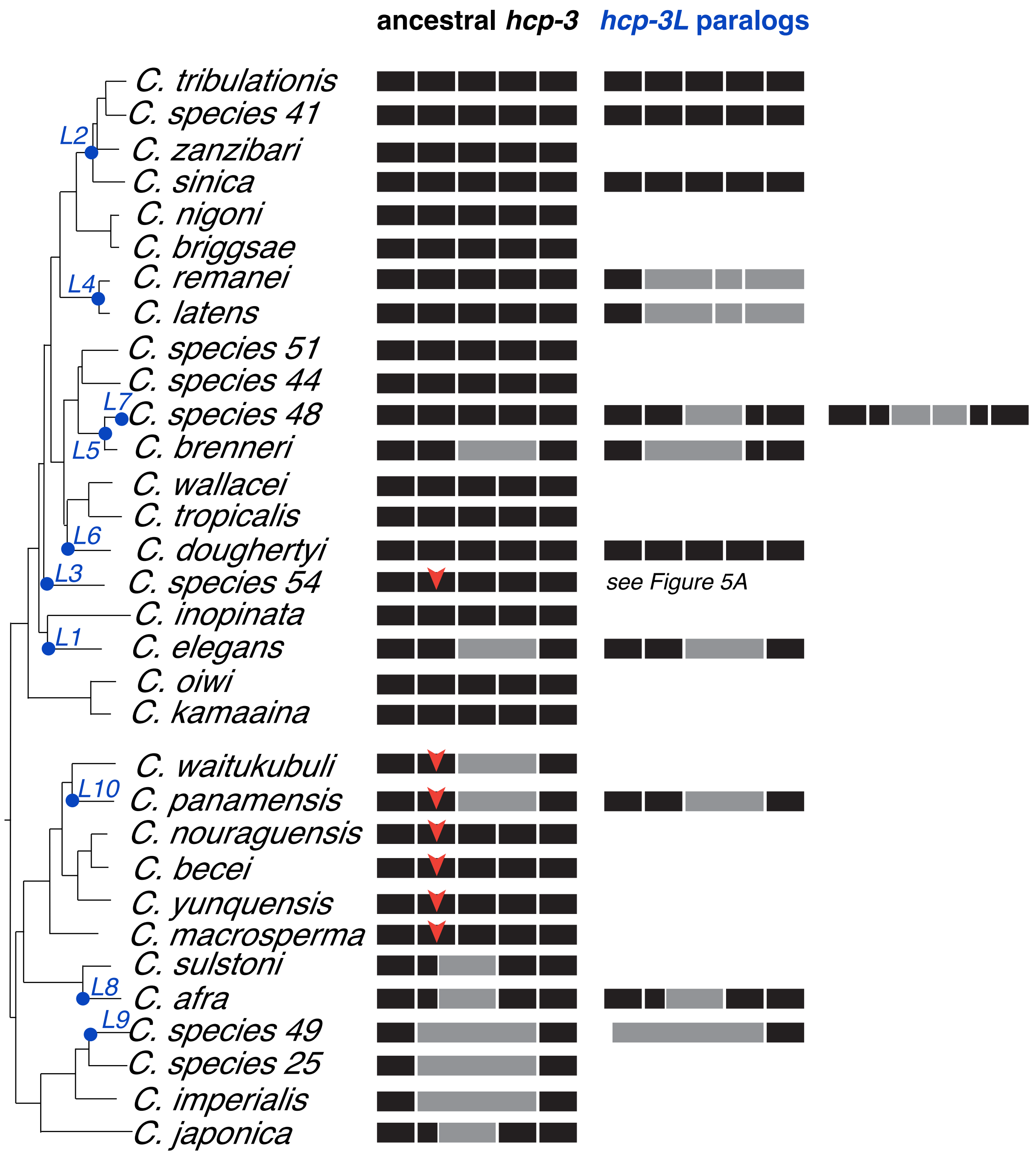

Supplementary Figure S1. Exon-intron junctions are largely retained in *Caenorhabditis hcp-3* and *hcp-3L* genes except for intron gains and losses that are likely a result of partial retro-transposition. Schematic of the exons of *hcp-3* and *hcp-3L* genes in *Caenorhabditis* species. Each box represents an exon with black boxes showing ancestral exons. Grey boxes depict exon fusion events that are a likely consequence of partial retrotransposition and overwriting of the genomic locus as has been previously observed (Robertson 1998; Cho, et al. 2004; Kiontke, et al. 2004). Red arrows indicate insertion events that likely create new introns. Exon duplications for *C. sp54 hcp-3* and *hcp-3L3* are discussed in more detail in Figure 5A in the main text. An N-terminal extension to *C. afra hcp-3L8* is not shown but discussed in more detail in Figure 5B.

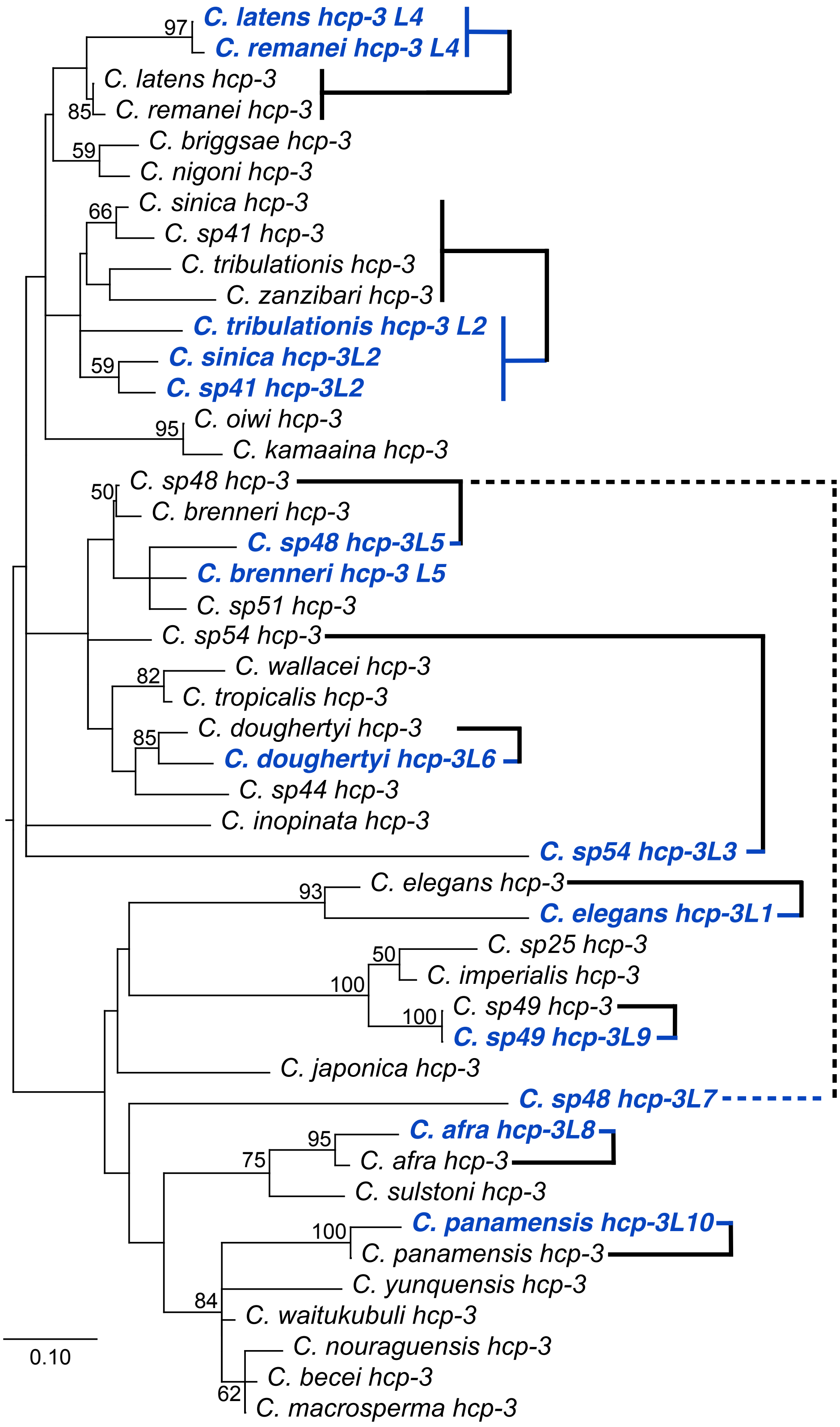

Supplementary Figure S2. Maximum-likelihood phylogenetic tree based on an amino acid alignment of HCP-3 and HCP-3L proteins encoded by *Caenorhabditis* species. A maximum likelihood tree based on an amino acid alignment of the histone fold domain (HFD) encoded by ancestral hcp-3 (black) and *hcp-3L* paralogs (blue) is shown as a phylogram (branch lengths are scaled to evolutionary divergence indicated). Bootstrap values of 40 and above are indicated. Overall, this phylogeny is much more poorly resolved than one based on the nucleotide alignment (Figure 2) and does not fully recapitulate known relationships between *Caenorhabditis* species or relationships between *hcp-3* and *hcp-3L* genes from the same species. However, well-resolved nodes agree between both the protein and nucleotide phylogenies and are also indicated in Figure 2.

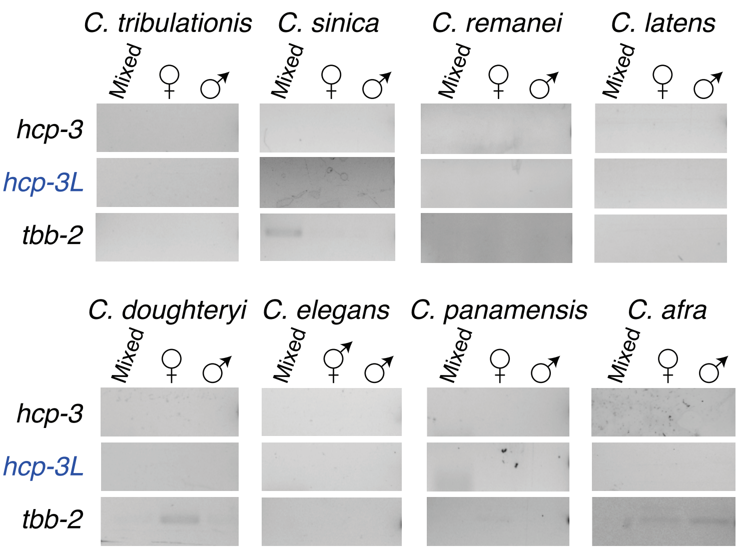

Supplementary Figure S3. RT-PCR controls for expression analysis of *hcp-3* and *hcp-3L* genes. No reverse transcriptase (-RT) PCR control of ancestral *hcp-3* (top), *hcp-3L* paralogs (middle), or *tbb-2* loading control genes (bottom) in selected species with *hcp-3L* duplicates. RNA from a mixed worm population of various larval stages, L4 or young adult females/hermaphrodites or L4 or young adult males were used. In some cases, we see very faint bands in the tbb-2 -RT controls compared to +RT samples.

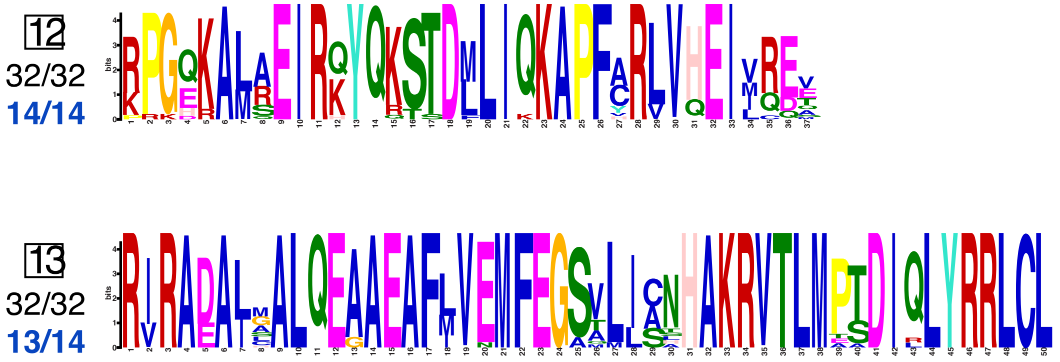

Supplementary Figure S4. Conserved motifs identified in the HCP-3 histone fold domains. Logo plots of conserved motifs 12 and 13, which reside in the HCP-3 histone fold domain. Proportion of all 32 ancestral HCP-3 proteins (black) or 14 HCP-3L duplicates (blue) that have retained the motifs are shown. All HCP-3 and HCP-3L proteins contain motifs 12 and 13, except for *C. sp54* HCP-3L3 which has a poor statistical match to motif 13 owing to high divergence (see Figure 5A).

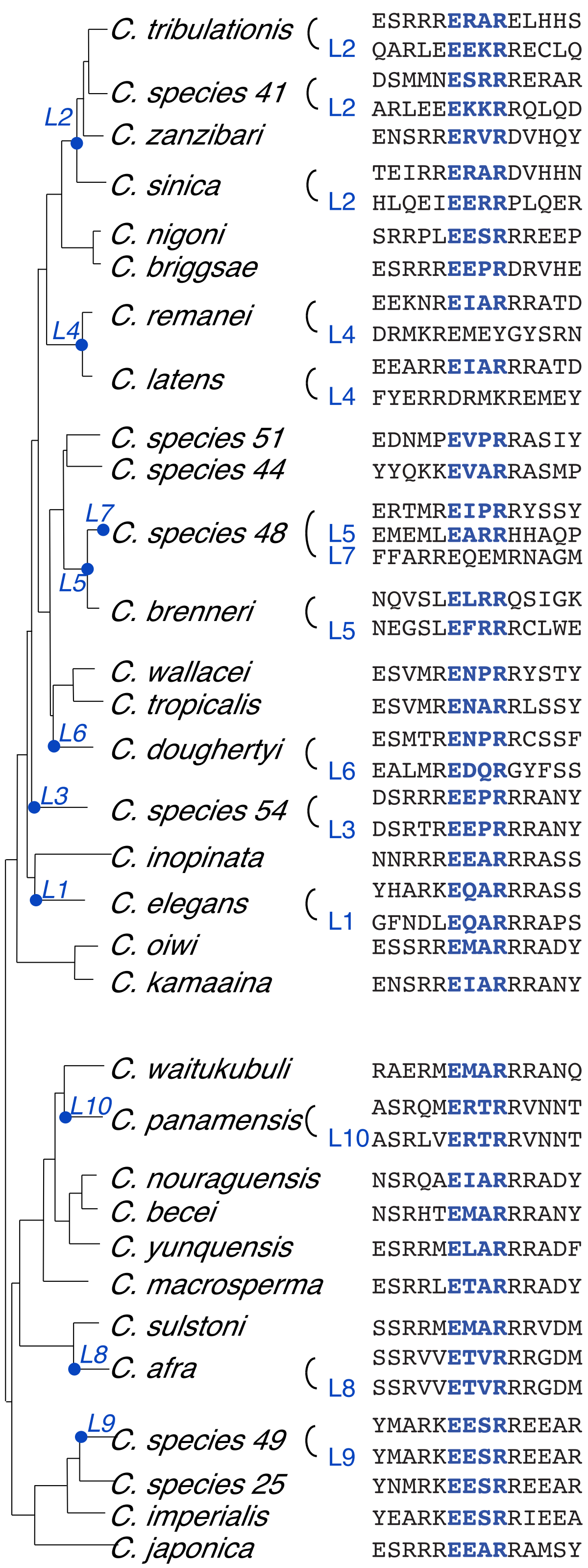

Supplementary Figure S5. The ExxR putative separase cleavage site is retained in most *Caenorhabditis* HCP-3 and HCP-3L proteins. Alignments of ExxR residues (blue colored) and flanking residues in HCP-3 and HCP-3L proteins are shown alongside a *Caenorhabditis* species tree.

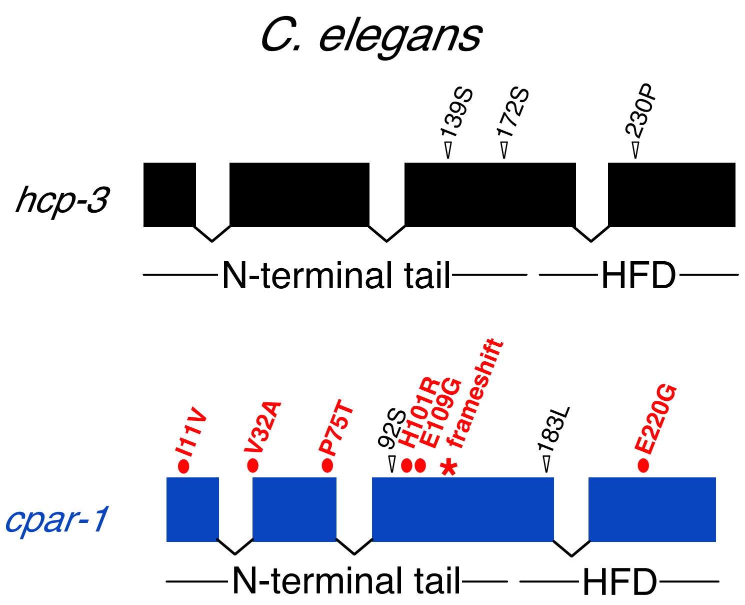

Supplementary Figure S6. A higher frequency of function-altering mutations is observed in *cpar-1* compared to *hcp-3* within natural *C. elegans* populations. Schematic of exon-intron structure of *hcp-3* (top, black) and cpar-1 (bottom, blue) coding regions in *C. elegans*, indicating the N-terminal domain and HFD. Natural variation found in *C. elegans* strains is indicated by arrowheads (black) and ovals (red) that represent synonymous and nonsynonymous mutations respectively. Three synonymous mutations and zero nonsynonymous mutations were found in *hcp-3* indicating extremely strong purifying selection, whereas six nonsynonymous and two synonymous mutations were found in *cpar-1*. In addition, a single nucleotide insertion in *cpar-1* that causes a frameshift resulting in an early stop codon was found.

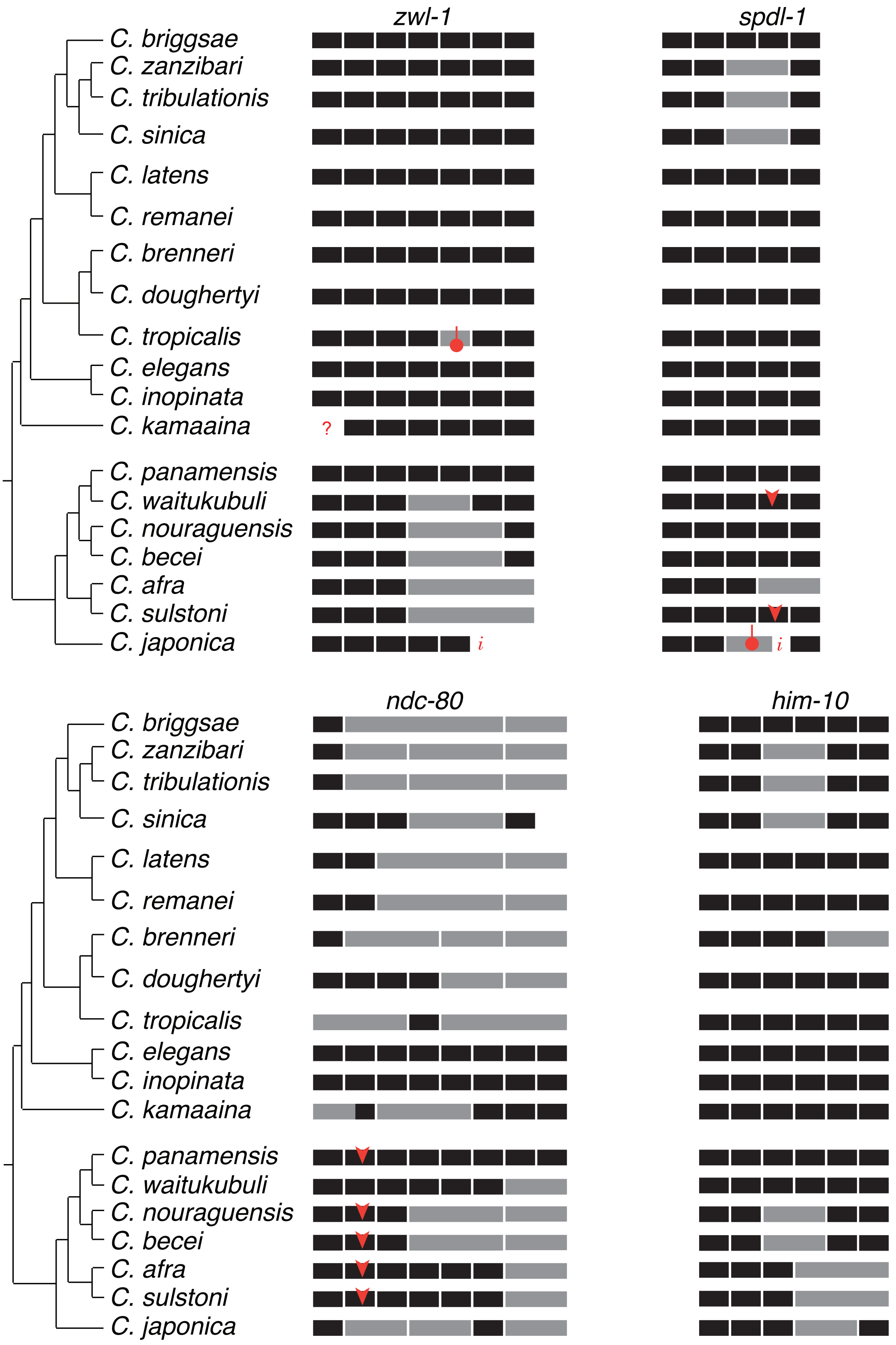

Supplementary Figure S7. Other kinetochore genes also display patterns of intron loss in *Caenorhabditis* species. Schematic of the exon arrangements of four genes (*zwl-1, spdl-1, ndc-80,* and *him-10*) encoding kinetochore proteins in a representative set of *Caenorhabditis* species. Each box represents an exon with black boxes showing ancestral exons and grey boxes showing fusion events between exons that likely arose due to partial retrotransposition and overwriting of the genomic locus (Robertson 1998; Cho, et al. 2004; Kiontke, et al. 2004). Red arrows indicate insertion events that likely create new introns and red dots represent deletions in exons. Incomplete genomic sequence information is indicated with an *'i'*. Although we were cannot identify exon 1 of *C. kamaaina zwl-1* using homology, it is unlikely to have been pseudogenized, since *zwl-1* is an essential gene and is present as an intact gene in all related species.
